# Changes in seasonal precipitation distribution but not annual amount affect litter decomposition in a secondary tropical forest

**DOI:** 10.1002/ece3.5635

**Published:** 2019-09-10

**Authors:** Shiqin Yu, Qifeng Mo, Yingwen Li, Yongxing Li, Bi Zou, Hanping Xia, Zhi'an Li, Faming Wang

**Affiliations:** ^1^ Key Laboratory of Vegetation Restoration and Management of Degraded Ecosystems South China Botanical Garden Chinese Academy of Sciences Guangzhou China; ^2^ University of Chinese Academy of Sciences Beijing China; ^3^ Xiaoliang Research Station for Tropical Coastal Ecosystems Chinese Academy of Sciences Maoming China; ^4^ College of Forestry and Architecture South China Agricultural University Guangzhou China

**Keywords:** C cycling, climate change, precipitation change, soil moisture, tropical forest

## Abstract

In the tropics of South China, climate change induced more rainfall events in the wet season in the last decades. Moreover, there will be more frequently spring drought in the future. However, knowledge on how litter decomposition rate would respond to these seasonal precipitation changes is still limited. In the present study, we conducted a precipitation manipulation experiment in a tropical forest. First, we applied a 60% rainfall exclusion in April and May to defer the onset of wet season and added the same amount of water in October and November to mimic a deferred wet season (DW); second, we increased as much as 25% mean annual precipitation into plots in July and August to simulate a wetter wet season (WW). Five single‐species litters, with their carbon to nitrogen ratio ranged from 27 to 49, and a mixed litter were used to explore how the precipitation change treatments would affect litter decomposition rate. The interaction between precipitation changes and litter species was not significant. The DW treatment marginally accelerated litter decomposition across six litter types. Detailed analysis showed that DW increased litter decomposition rate in the periods of January to March and October to December, when soil moisture was increased by the water addition in the dry season. In contrast, WW did not significantly affect litter decomposition rate, which was consistent with the unchanged soil moisture pattern. In conclusion, the study indicated that regardless of litter types or litter quality, the projected deferred wet season would increase litter decomposition rate, whereas the wetter wet season would not affect litter decomposition rate in the tropical forests. This study improves our knowledge of how tropical forest carbon cycling in response to precipitation change.

## Graphical abstract

Tropical forest litter decomposition was affected by seasonal precipitation changes but not the increased annual amount.

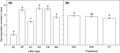

## 1 INTRODUCTION

Litter decomposition is a key process of turning carbon (C) and nutrients from organic to inorganic state, which could be utilized by plants and microbes in terrestrial ecosystems. The intensified hydrological cycle caused by global warming has resulted in global and regional precipitation changes (Huntington, 2006; Min, Zhang, Zwiers, & Hegerl, 2011; Seneviratne et al., 2010). A large number of studies have revealed that precipitation is among the most important factors regulating litter decomposition rate in terrestrial ecosystems (Aerts, 1997; Taylor et al., 2017; Wieder, Cleveland, & Townsend, 2009). As a result, knowledge on how litter decomposition rate responds to precipitation changes would contribute to a better prediction of C and nutrient cycles in terrestrial ecosystems.

Litter decomposition is a series of physical and chemical breakdowns of plant detritus including leaching, fragmentation, and chemical alteration. Precipitation change can affect litter decomposition through physical leaching (Cleveland, Wieder, Reed, & Townsend, 2010; Currie & Aber, 1997; Deng et al., 2018), thermal balance changes (Lagergren & Lindroth, 2002; Maes & Steppe, 2012), and soil water availability (Beier et al., 2012; Knapp et al., 2008; Kramer & Boyer, 1995). The changes in soil water availability would affect the abundance and community of soil microbes (Fierer, Schimel, & Holden, 2003; Manzoni, Schimel, & Porporato, 2012; Wagener & Schimel, 1998) and soil fauna (Lindberg, Engtsson, & Persson, 2002; Pritchard, 2011; Taylor, Schroter, Pflug, & Wolters, 2004), thus exerting considerable influences on litter decomposition rate (Handa et al., 2014; Hattenschwiler, Tiunov, & Scheu, 2005; Meier & Bowman, 2008).

Most of our knowledge of the precipitation change effect on litter decomposition rate derives from the comparisons among sites along natural precipitation gradients or precipitation amount manipulation experiments (e.g., Campos, Germino, & Graaff, 2017; Powers et al., 2009; Wieder et al., 2009). Generally, litter decomposition rate would increase with precipitation amount in temperate grasslands and forests (Campos et al., 2017; Gaxiola & Armesto, 2015; Santonja et al., 2017; Zheng, Guo, Li, Zhang, & Han, 2017), whereas litter decomposition rate would decrease with increasing precipitation in humid tropical forests, mostly because excessive water depressed the activity of decomposers (Schuur, 2001). However, precipitation would change not only in annual amount, but also in seasonal distribution (IPCC, 2007, 2013). At present, there are many pieces of evidence suggesting changing seasonal precipitation pattern in tropical forests (Chadwick, Good, Martin, & Rowell, 2016; Greve et al., 2014). In the tropics of South China, previous studies reported that the wet season was coming late and precipitation amount in wet season becomes larger (Fang, Piao, He, & Ma, 2004; Luo et al., 2008; Zhou et al., 2011). Seasonal precipitation changes have been reported to have a different effect as changes in precipitation amount on sap flow (Zeppel, Macinnisng, Ford, & Eamus, 2008) and plant photosynthesis (Volder, Briske, & Tjoelker, 2013). However, there is still limited report on how seasonal precipitation change would affect litter decomposition rate in tropical forests.

Litter traits, such as C:N ratio, strongly regulate litter decomposition rate (Aerts, 1997; Chapin, Matson, & Vitousek, 2011; Cornwell et al., 2008) and its response to environmental changes (Knorr, Frey, & Curtis, 2005; Liu et al., 2017). There were some studies suggesting that the effect of precipitation changes on litter decomposition rate varied with litter quality (e.g., Austin & Vitousek, 2000; Sanaullah, Rumpel, Charrier, & Chabbi, 2012; Santonja et al., 2017; Suseela, Tharayil, Xing, & Dukes, 2013). However, results on how litter quality regulates the response of litter decomposition rate from previous studies sometimes contradicted to each other. For example, Liu, Huang, Han, Sun, and Zhou (2006) found that increased precipitation accelerated decomposition of high‐quality litter, whereas Wang, Xu, et al. (2017) reported that increased precipitation enhanced decomposition of low‐quality litter. Therefore, the site‐specific knowledge on how litter quality regulates their decomposition response to precipitation changes is needed.

In this study, we established a precipitation manipulation experiment through rainfall exclusion or/and water addition to simulate the projected deferred wet season and wetter wet season in a tropical forest. We used five single‐species litters and their mixture to conduct a litterbag decomposition experiment. The primary aim of this study was to explore how litter decomposition rate would be affected by these seasonal precipitation changes. In this experimental site, we have observed that the deferred wet season (DW) treatment can increase soil moisture in dry season, whereas the wetter wet season (WW) did not significantly affect soil moisture (Yu et al. under review). Therefore, we hypothesized that DW would accelerate litter decomposition, whereas WW would have no effect on litter decomposition rate. Additionally, we also hypothesized that the response of litter decomposition rate to the precipitation change treatments would be species‐specific as these litters differed in litter quality.

## 2 MATERIALS AND METHODS

### 2.1 Study sites

The precipitation manipulation experiment was conducted at the Xiaoliang Tropical Coastal Ecosystem Research Station (110°54′E, 21°27′N), Chinese Academy of Sciences, Guangdong Province, China. The climate here is a tropical climate with a distinct wet (from April to September) and dry season (from October to March). The mean annual temperature is 23°C, and the mean annual precipitation is 1,400–1,700 mm, respectively. The soil is lateritic and developed from deeply weathered granite (Wang, Ding, et al., 2017). Our experimental site was located in a secondary tropical forest. The forest started as *Eucalyptus exserta* plantation in 1959, and then, 312 native tree species were introduced in the 1960s (Ding, Yi, Liao, Martens, & Insam, 1992; Ren et al., 2007). According to the survey performed in 2015, the dominant tree species are *Aphanamixis polystachya*, *Schefflera octophylla*, *Carallia brachiate*, *Symplocos chunii*, *Acacia auriculaeformis*, *Photinia benthamiana*, and *Cinnamomum burmanni*, the dominant shrub and herb species are *Dicranopteris dichotoma*, *Lygodium japonicum*, *Blechnum orientale*, *Psychotria rubra*, *Uvaria microcarpa*, and *Clerodendrum cyrtophyllum*.

### 2.2 Experimental design

In 2012, we established four experimental blocks in the tropical forest. Each experimental block consisted of a deferred wet season (DW), a wetter wet season (WW), and a control plot (CT). Each plot was 12 m × 12 m and at least 3 m away from each other. In DW and WW, polyvinyl chloride (PVC) plates were inserted into the depth of 0.5 m along each plot's borders to prevent surface runoff and lateral movement of water from/into the surrounding soil.

In each DW plot, we used many pieces of clear, soft, and photosynthetically active radiation transmitting greenhouse plastic sheet to construct a rainout shelter for partial rainfall exclusion. Each pieces of plastic sheet was mounted on a stainless steel frame approximately 2.5 m above the ground. The plastic sheet could be unfolded or folded on the stainless steel frame. In the first two months of the wet season (April and May), the plastic sheet was unfolded, which made it U shape for rainfall interception. All the unfold sheet in total covered 60% of the ground area of the DW plots. As a result, 60% of rainfall would be intercepted and runoff from the DW plots along the sheet by gravity. By doing this, the onset of wet season was deferred for 2 months. During the rest of the year, the plastic was folded, which made it I shape, thus having little effect on the precipitation.

During October to November, water was added into DW plots once a week through an understory sprinkling system. The understory sprinkling system consisted of nine sprayers distributing uniformly in each plot. The sprayer was 1 m height and connected to water pipelines. The water used for sprinkling was ground water from a nearby deep well. In total, eight times of water addition were conducted and the amount of water added into the DW plots equaled the 60% of rainfall in CT. The water addition was conducted to delay the end of the wet season in DW plots.

In WW plots, 50 mm of water was added every week in July and August through an understory sprinkling system similar to that in DW plots. Water additions in WW were conducted to simulate an approximately 25% increase in annual precipitation in the wet season.

Precipitation manipulations in DW and WW started in 2013, and the same manipulations continued in 2014 and 2015. During the experimental period, CT plots received ambient precipitation inputs.

### 2.3 Litter decomposition

Leaf litter of five dominant tree species, including *S. chunii* (SC), *A. polystachya* (AP), *Acacia crassicarpa* (AC), *Schefflera octophyllaII* (SO), and *Carallia brachiate* (CB), were collected using litter traps around the experimental site. They were taken back to the laboratory and air‐dried. To measure the water content air‐dried litters, they were dried at 60°C until reached a constant weight. Six litter types, including five single‐species litters and a litter mixture of the five species, were prepared for field decomposition using 2.0 mm mesh litterbag (25 cm × 25 cm) with mesh size designed to allow colonization by microbes and most of soil fauna. For each single‐species litter, 10 g of air‐dried single‐species leaf litter was placed into a litterbag; the mixed litter consisted of every litter species of 2.0 g. In each plot, two subplots were established. The subplot was at least 3 m away from each other and the plot border. In each subplot, six bags of each litter type were placed on the forest floor surface. A total of 864 litterbags were placed in the experiment site in early January of 2015. In late March, late June, early October, and late December 2015, one bag for each litter type in each subplot was taken back to laboratory. In total, litterbags were collected four times, and the decomposition was artificially divided into four decomposition course. Litters taken back to laboratory were carefully brushed to prevent soil contamination. After that, they were dried at 60°C until a constant weight. Dry litters were weighed to determine the residual mass.

### 2.4 Litter and soil analysis

The organic C and total N content were analyzed with fresh litter. Initial litter organic C content was determined with the wet‐combustion method. Litter total N content was determined by micro‐Kjeldahl digestion method and then measured colorimetrically by FIA (Lachat Instruments, USA). Soil moisture (volumetric water content) was determined in field about 2–3 times a month using a soil moisture meter with a FDS‐100 sensor (Uni2000; Beijing Unism Technologies, Inc.).

### 2.5 Data analysis

Data transformation was done to meet the assumptions of normality and homogeneity of variances when it was necessary. We used a repeated measure ANOVA (RM‐ANOVA) to examine the effect of treatments on soil moisture in each decomposition course. One‐way ANOVA was conducted to test the difference of initial litter characteristics among six litter types. The litter decomposition constant (*k*) was calculated according to: *k* = ln(*M_t_*/*M*_0_)/*t*, where *M_t_* is the final litter mass, *M*_0_ is the initial litter mass converted to the equivalent mass at 60°C, and *t* is the incubation time (in years). Mean mass loss rate in each decomposition course was calculated by dividing the mass loss with days in the decomposition course. Two‐way ANOVAs were used to test for the effects of litter type, precipitation change, and their interactions on *k* over the whole experiment and on litter mass loss rate in decomposition course. Multiple comparisons were conducted using an LSD method after ANOVAs. All statics analyses were conducted in SPSS 20.0 (Armonk, NY: IBM Corp).

## 3 RESULTS

### 3.1 Soil moisture

**Figure 1:**
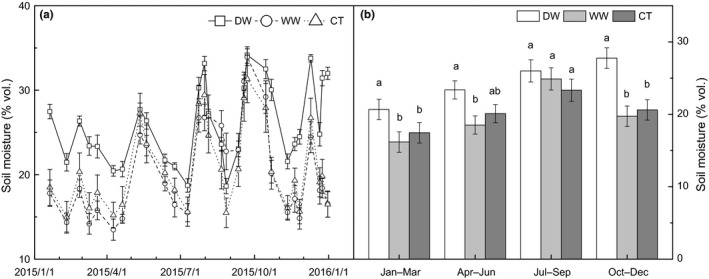
Temporal dynamic of soil moisture (a) and mean soil moisture in each decomposition course (b) in the deferred wet season (DW), wetter wet season (WW), and control (CT) plots. Different letters represent statistically significant differences (LSD multiple comparison tests at *p* < .050)

According to the time of litterbag collection, the decomposition was artificially divided into four courses. In the period of January to March, soil moisture in DW was higher than that in CT (Figure 1a), which could be attributed to the water addition in October and November of 2014. The mean soil moisture in this course in DW was 20.7%, which was 18.5% higher than soil moisture in CT (*p* = .004) (Figure 1b). The difference between DW and CT in the periods of April–June and July–September became smaller (Figure 1a). The mean soil moisture in DW was 16.3% (*p* = .099) and 11.4% (*p* = .250) higher than CT in the periods of April to June and July to September, respectively. After the water addition in October and November, the difference of soil moisture between DW and CT increased (Figure 1a), and the soil moisture in DW was significantly higher than that in CT (*p* = .006) in the period of October to December (Figure 1b).

In contrast, the WW treatment had limited effects on soil moisture (Figure 1a,b). In the period of July to September, the water addition in WW seemed to increase soil moisture, especially in October (Figure 1a), but, overall, the effect was not significant (*p* = .495).

### 3.2 Initial litter quality

Litter quality was indicated by the ratio of litter organic C to total N content (C:N ratio). Initial litter organic C (*p* < .001) and total N content (*p* < .001) and the C:N ratio (*p* < .001) varied significantly among six litter types (Table 1). The *S. chunii* had the highest C:N ratio as it had highest organic C content but lowest total N content.

**Table 1:** Initial organic carbon (C) content, total nitrogen (N) content, and C:N ratio for litters of *Symplocos chunii* (SC), *Aphanamixis polystachya* (AP), *Acacia crassicarpa* (AC), *Schefflera octophylla* (SO), *Carallia brachiate* (CB), and mixed litter (Mix)

Litter type	Organic C content (%)	Total N content (mg/g)	C:N ratio
SC	62.38 (1.05)^a^	12.83 (0.34)^c^	48.65 (0.50)^a^
AP	50.91 (1.10)^b^	16.05 (0.46)^c^	31.73 (0.46)^b^
AC	46.18 (0.76)^c^	22.11 (0.51)^a^	20.90 (0.17)^d^
SO	52.45 (1.08)^b^	16.93 (0.41)^b^	31.02 (0.96)^b^
CB	41.80 (0.65)^d^	15.55 (0.62)^b^	26.98 (1.24)^c^
Mix	50.41 (0.85)^b^	16.45 (0.26)^b^	30.67 (0.98)^b^

Note

Different letters represent statistically significant differences (LSD multiple comparison tests at *p* < .050).

### 3.3 Litter decomposition constant *k*

**Figure 2:**
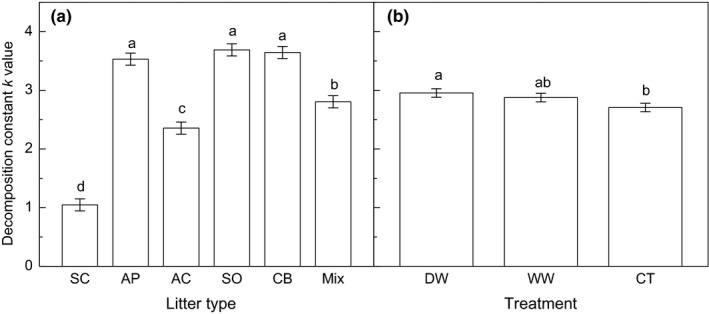
The effects of litter type (a) and precipitation change (b) on litter decomposition constant *k*. AC, *Acacia crassicarpa*; AP, *Aphanamixis polystachya*; CB, *Carallia brachiate*; CT, control; DW, deferred wet season; Mix, mixed litter; SC, *Symplocos chunii*; SO, *Schefflera octophylla*; WW, wetter wet season. Different letters represent statistically significant differences (LSD multiple comparison tests at *p* < .050)

The decomposition constant *k* of six litter types across treatments ranged from 0.99 to 3.91 (*p* < .001, Table 2, Figure 2a). Although two‐way ANOVA only detected a marginally significant difference among treatments (*p* = .078), the multiple comparisons indicated that DW significantly increased *k* (*p* = .028), whereas WW tended to increase *k* (*p* = .128, Figure 2b). The litter type did not significantly interact with the treatments on influencing litter decomposition constant (*p* = .974, Table 2). In contrast, WW tended to increase *k* of all litter types, but not significantly (Figure 2b).

**Table 2:** Two‐way ANOVA examining main and interactive effects of litter type and precipitation change on litter decomposition constant *k*

Variance of source	*df*	*F*	Sig.
Litter type	5	88.324	<0.001
Precipitation change	2	2.679	0.078
Litter type × Precipitation change	10	0.316	0.974

### 3.4 Litter mass loss in each period

**Figure 3:**
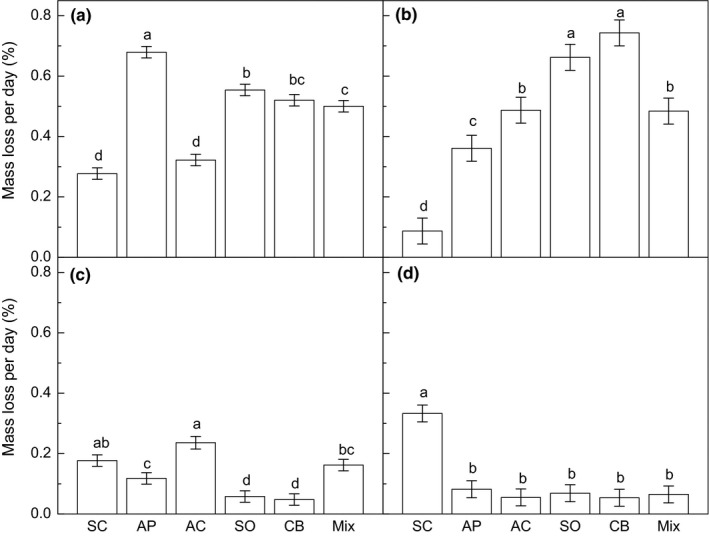
The effect of litter type on litter mass rate in the periods of January to March (a), April to June (b), July to September (c), and October to December (d). AC, *Acacia crassicarpa*; AP, *Aphanamixis polystachya*; CB, *Carallia brachiate*; Mix, mixed litter; SO, *Schefflera octophylla*; SC, *Symplocos chunii*. Different letters represent statistically significant differences (LSD multiple comparison tests at *p* < .050)

**Figure 4:**
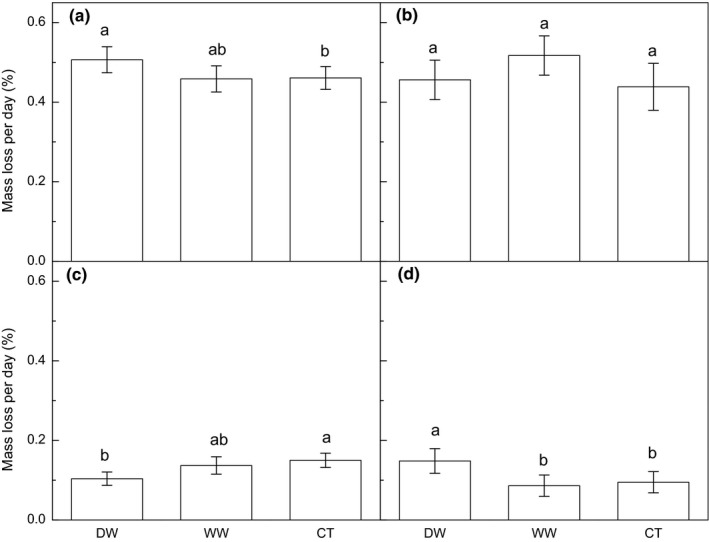
The effect of precipitation change on litter mass rate in the periods of January to March (a), April to June (b), July to September (c), and October to December (d). CT, control; DW, deferred wet season; WW, wetter wet season. Different letters represent statistically significant differences (LSD multiple comparison tests at *p* < .050)

Litter mass loss rate varied significantly among litter types in each course (*p* < .001, Table 3, Figure 3). The treatment significantly affected mass loss in the periods of January–March (*p* = .022) and July–September (*p* = .036), and tended to affect mass loss rate in October–December (*p* = .059). Multiple comparisons after the two‐way ANOVA showed that DW increased mass loss rate in the periods of January to March (*p* = .019, Figure 4a) and October to December (*p* = .028, Figure 4d), but decreased mass loss rate in the period of July to September (*p* = .021, Figure 4c). In contrast, WW tended to increase the mass loss rate in the period of April–June (*p* = .069, Figure 4d). We did not find a significant interactive effect between litter type and treatment in any course (Table 3).

**Table 3:** Two‐way ANOVA examining main and interactive effects of litter type and precipitation change on litter mass loss rate in each decomposition course

	Variance of source	*df*	*F*	Sig.
January–March	Litter	5	60.162	<0.001
	Precipitation change	2	3.91	0.026
	Litter × Precipitation change	10	0.764	0.662
April–June	Litter	5	28.143	<0.001
	Precipitation change	2	1.779	0.178
	Litter × Precipitation change	10	0.646	0.768
July–September	Litter	5	11.948	<0.001
	Precipitation change	2	3.531	0.036
	Litter × Precipitation change	10	0.646	0.768
October–December	Litter	5	14.878	<0.001
	Precipitation change	2	2.778	0.071
	Litter × Precipitation change	10	0.605	0.803

## 4 DISCUSSION

### 4.1 The effect of seasonal precipitation changes on litter decomposition

In the present study, the decomposition constants for six litter types ranged from 0.99 to 3.91. The decomposition constants were comparable to those reported in tropics (Aerts, 1997; Cleveland, Reed, & Townsend, 2006). The present study showed that a wetter wet season would not significantly affect litter decomposition rate. Though precipitation changes may affect litter decomposition rate through litter dissolved matter leaching or soil temperature, the role of soil moisture is believed to be predominant in regulating the ecosystem responses under precipitation changes (Beier et al., 2012; Knapp et al., 2008; Kramer & Boyer, 1995). Previous studies showed that the effect of precipitation changes on litter decomposition rate was tightly correlated with the alteration of soil moisture (e.g., Campos et al., 2017; Cornejo, Varela, & Wright, 1994; Schuur, 2001). For example, a previous study in a desert found that 30% increase in annual precipitation in summer or winter did not change soil moisture and did not alter litter decomposition rate either (Zhao, Huang, Ma, Li, & Zhou, 2012). In this study, WW did not significantly affect soil moisture, which may explain unchanged decomposition rate.

In contrast, DW treatment significantly increased the soil moisture in the dry season and enhanced the decomposition rate across six litter types (Figures 1 and 3). Specifically, the water addition in October and November increased soil moisture, and the effect sustained until March, the beginning of the wet season. Increased soil moisture can improve the activity of soil decomposers (Fierer et al., 2003; Manzoni et al., 2012; Pritchard, 2011) and further accelerated the litter decomposition. This was consistent with our result that DW increased soil microbial biomass indicated by PLFAs (Figure S1a). In this study, DW significantly increased mass loss in the periods of January to March and October to December, when soil moisture was significantly increased. The results thus were consistent with previous precipitation manipulation studies suggesting that litter decomposition rate increased with higher soil moisture (Campos et al., 2017; Gaxiola & Armesto, 2015). For example, Vasconcelos, Zarin, da Rosa, de Assis Oliveira, and de Carvalho (2007) found that litter decomposition rates were up to 2.4 times higher in irrigated plots than in control plots in a dry season irrigation experiment in eastern Amazonian forest. Similarly, in a seasonally dry tropical forest in Panama, Wieder and Wright (1995) found dry season irrigation reduced forest floor litter mass throughout the year. These studies combined with ours indicated that litter decomposition rate was limited by soil water availability in dry season in these tropical forests.

### 4.2 The effect of seasonal precipitation change was independent on litter quality

Litter C:N ratio is widely used to refer to litter quality (Enríquez, Duarte, & Sand‐Jensen, 1993; Gholz, Wedin, Smitherman, Harmon, & Parton, 2000). In the present study, the litter C:N ratio varied widely from 27 to 49, which has resulted in the high variation in litter decomposition rate. However, contrasted to our second hypothesis, the effect of the precipitation changes on litter decomposition rate was independent on litter quality in this study. Most of previous studies supporting a litter quality‐dependent response of decomposition rate to precipitation changes were conducted in temperate ecosystems (e.g., Liu et al., 2006; Santonja et al., 2017; Wang, Xu, et al., 2017), with limited reports in tropics. Due to the different environmental factors and evolutionary history (Hawkes & Keitt, 2015; Willis, Jeffers, & Tovar, 2018), C cycling response to precipitation changes generally depended on ecosystem types (Liu et al., 2016; Wu, Dijkstra, Koch, Penuelas, & Hungate, 2011). Our results were in agreement with a previous study in tropical forests, which showed that enhanced precipitation increased the decomposition rate of all litters, no matter which species it is (Austin & Vitousek, 2000).

Few studies investigated the underlying mechanism contributing to the different responses to precipitation changes between different litters. In a European grassland, Sanaullah et al. (2012) found that the decomposition of a lignin‐rich litter was more depressed by drought than lignin‐poor litter, which was attributed to a reduction in lignin degradation. This was consistent with some previous studies, which found nitrogen enrichment inhibited the decomposition of high‐lignin litters by depressing lignin decaying (Carreiro, Sinsabaugh, Repert, & Parkhurst, 2000; Knorr et al., 2005). Fungi play a critical role in lignin degradation (Kirk & Farrell, 1987; Osono, 2007). In the present study, DW increased both the fungal and bacterial biomass, but did not affect the soil fungi to bacteria ratio (Figure S1b–d). The results may suggest that DW consistently increase the degradation of all kinds of litter organic matter.

Previous studies which observed quality‐dependent responses to precipitation changes typically used litterbags of mesh size not larger than 1.0 mm (e.g., Liu et al., 2006; Sanaullah et al., 2012; Wang, Xu, et al., 2017), which excluded meso‐ and macrofauna in the litter decomposition (Bradford, Tordoff, Eggers, Jones, & Newington, 2002; Setala, Marshall, & Trofymow, 1996). Tropical forests have a high diversity of soil fauna (see Table S1 for the soil biota in the tropical forest), which can make a considerable contribution to litter decomposition (Garcia‐Palacios, Maestre, Kattge, & Wall, 2013; Wall et al., 2008). Soil fauna is sensitive to precipitation changes (Lindberg et al., 2002; Pritchard, 2011; Taylor et al., 2004). As a result, the use of small mesh size litterbag would miss the effect of changed soil biota induced by precipitation changes on litter decomposition. The participation of soil fauna could result in the quality‐independent response of litter decomposition rate to environmental changes (Knorr et al., 2005). For example, Riutta et al. (2012) showed that the presence of macrofauna reduced the difference of decomposition rate between two contrasting litters under watering treatments. As a result, we suggest that the litterbag of 2 mm mesh size could allow more soil fauna to take part in the litter decomposition in the present study, which resulted in a consistent response of decomposition rate to the precipitation changes among litters with different quality.

## CONFLICTS OF INTEREST

The authors declare that they have no conflict of interests.

## AUTHOR CONTRIBUTIONS

F.W., S.Y., and Z.L. planned and designed the experiment. Q.M., B.Z., Y.L., Y.L., H.X., and S.Y. conducted the field work. F.W. and S.Y. analyzed data. S.Y. made the first version of the draft. F.W. and S.Y wrote the final version of manuscript based on the comments of all other coauthors.

## Data Availability

Soil microbial phospholipid fatty acids data are provided in Figure S1, and soil fauna community data are provided in Table S1.
