# Supplementary material for: Changes in seasonal precipitation distribution but not annual amount affect litter decomposition in a secondary tropical forest
Source: Ecol Evol. 2019 Sep 10;9(19):11344–52. doi: 10.1002/ece3.5635 (PMC6802026; doi:10.1002/ece3.5635)
Supplement: Supplementary file 1 [file ECE3-9-11344-s001.docx]

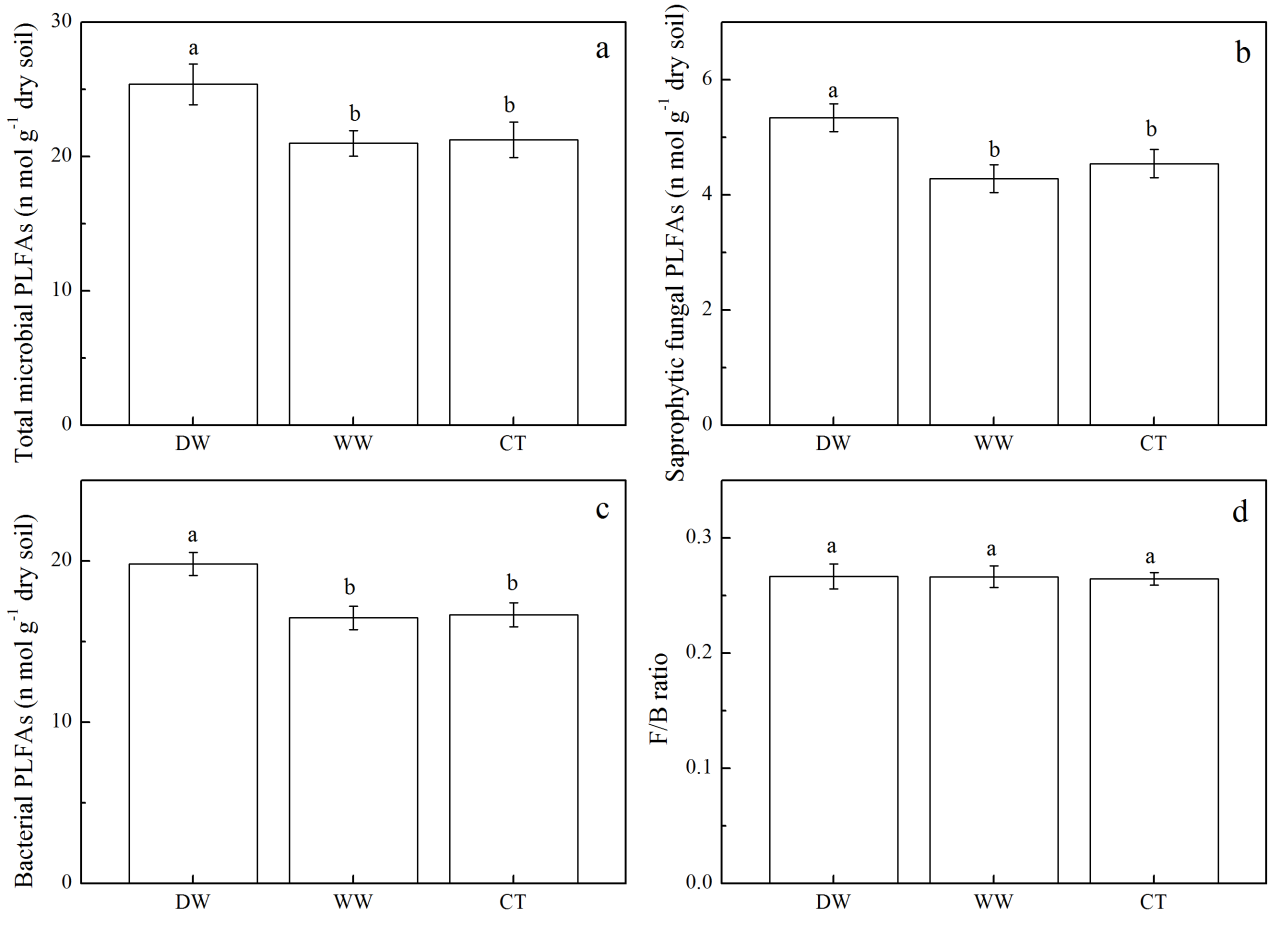


Figure S1 The total soil microbial phospholipid fatty acids (PLFAs) (a), saprophytic fungal PLFAs (b), bacterial PLFAs (c) and the ratio of fungal to bacteria PLFAs (d) in the deferred wet season plots (DW), wetter wet season (WW) and control (CT) plots in 2015. The extraction of soil microbial PLFAs and calculation of each group of soil microbe followed [Li *et al.* (2015)](#_ENREF_1). Different letters represent statistically significant differences (Least significant difference multiple comparison tests at *p* < 0.050)

Table S1 Soil animal density in the forest floor of the tropical forest in 2016. The soil animals were extracted in a modified Tullgren funnel (2 mm mesh size) for 48 hours.

| Soil animal | Density (individual 100 g^-1^ dry litter) | | |
| --- | --- | --- | --- |
| Acariformes | | 1123.5 |  |
| Collembola | | 116.6 |  |
| Parasiformes | | 44.6 |  |
| Coleoptera | | 44.6 |  |
| Isopoda | | 29.8 |  |
| Diptera | | 27.3 |  |
| Araneae | | 14.9 |  |
| Psocoptera | | 12.4 |  |
| Hymenoptera | | 9.9 |  |
| Enchytraeidae | | 7.4 |  |
| Blattoptera | | 5.0 |  |
| Thysanoptera | | 5.0 |  |
| Lepidoptera | | 5.0 |  |

| Polyzonida | 2.5 |
| --- | --- |
| Homoptem | 2.5 |

**Reference**

Li, J., Li, Z., Wang, F., Zou, B., Chen, Y., Zhao, J., Mo, Q., Li, Y., Li, X. & Xia, H. (2015) Effects of nitrogen and phosphorus addition on soil microbial community in a secondary tropical forest of China. *Biology and Fertility of Soils,* **51,** 207-215.
